# SARS-CoV-2 Antibody Seroprevalence in Patients With Cancer on Systemic Antineoplastic Treatment in the First Wave of the COVID-19 Pandemic in Portugal

**DOI:** 10.7759/cureus.22428

**Published:** 2022-02-21

**Authors:** Gonçalo Fernandes, Paulo Paixão, Laura Brum, Teresa Padrão, Jorge Correia, Joana Albuquerque, Catarina Pulido, Mónica Nave, Teresa Timóteo, Tânia Rodrigues, Filipe Costa, José L Passos-Coelho

**Affiliations:** 1 Medical Oncology, Hospital da Luz Lisboa, Lisboa, PRT; 2 Laboratory Medicine, Hospital da Luz Lisboa, Lisboa, PRT; 3 Laboratory Medicine, Synlab Portugal, Lisboa, PRT; 4 Oncology, Hospital da Luz Lisboa, Lisboa, PRT; 5 Management, Hospital da Luz Lisboa, Lisboa, PRT

**Keywords:** cancer, oncology, seroprevalence study, serology, sars-cov-2, covid-19

## Abstract

At the time of the first wave of the COVID-19 pandemic, patients with cancer were considered to be at high risk of serious illness and had a higher exposure risk since they needed frequent and nondeferrable hospital visits. Serological tests were not routinely used, and seroprevalence in this population was unknown.

A single-center, cross-sectional study was developed to determine the seroprevalence of anti-SARS-CoV-2 antibodies (Abs) in patients with cancer undergoing systemic antineoplastic treatment. One hundred patients were consecutively recruited in a two-week period (6th-20th May 2020), and serum samples were tested for the presence of immunoglobulin M (IgM) and immunoglobulin G (IgG) Abs directed against both spike (S) and nucleocapsid (N) SARS-CoV-2 proteins in two distinct time points (at recruitment and 4-8 weeks later). IgG-positive results were subject to confirmation, in the same serum sample, using two distinct assays.

At the time of the first study visit, no patient had a previously confirmed diagnosis of COVID-19, one reported previous contact with a COVID-19 patient, and all had a baseline SARS-CoV-2-negative RT-PCR. Two patients tested positive for SARS-CoV-2 IgG in the first study visit, which was not confirmed in either of the two confirmatory assays. Seventy-two patients were tested at the second study visit, all with negative IgG tests. IgM was persistently positive at both study visits in one patient and was positive in another patient at the second study visit, both with negative RT-PCR and serum IgG. No patient tested positive for RT-PCR within the study timeframe.

No evidence of prior or acute SARS-CoV-2 infection was documented in this cohort of patients with cancer undergoing systemic treatment, and no additional exposure risk was documented compared to general population seroprevalence studies. The study was inconclusive regarding the role of SARS-CoV-2 serology in patients with cancer in the early phase of the pandemic. This study did show that, with adherence to recommended preventive measures, it was safe to maintain systemic cancer therapy.

## Introduction

The majority of patients with COVID-19 develop antibodies (Abs) against SARS-CoV-2 [1]. While the gold standard for acute COVID-19 diagnosis remains the detection of SARS-CoV-2 virus in respiratory tract swab specimens by RT-PCR [2], serological tests detecting Abs, immunoglobulin G (IgG), and immunoglobulin M (IgM) may identify patients who have been infected in the past, including prior asymptomatic infections, and can be used to measure herd immunity to the disease [3].

Available scientific evidence at the time of the first COVID-19 pandemic wave indicated a worse prognosis of the disease in patients with cancer, with early reports from China showing that the overall case-fatality rate was 2% in the general population and 5.6% in patients with preexisting cancer [4], and in one cohort, the 30-day mortality rate reached 29% [5]. Since April 2020, the Portuguese National Health Authority recommendations determined that molecular nucleic acid amplification tests for SARS-CoV-2 detection in upper respiratory tract swab specimens should be performed prior to each treatment cycle in patients with cancer undergoing chemotherapy, even in asymptomatic patients [6]. Serological tests were not routinely used at that time, and there was scarce available data on seroprevalence in this patient population.

Patients with cancer needed nondeferrable hospital visits, both for evaluation and for treatment, despite the general population’s “stay at home” practice. We hypothesized that these patients could be at a greater exposure risk, and we developed a cross-sectional study to determine the seroprevalence of anti-SARS-CoV-2 antibodies (IgM and IgG) at two distinct time points during the first wave of COVID-19 pandemic in patients with cancer (solid tumors or hematological cancer) undergoing systemic antineoplastic treatment in our Oncology Unit.

This article was previously posted to the medRxiv preprint server on 2nd February 2022.

## Materials and methods

Study design

The study included two outpatient visits. A two-visit design was used to minimize the risk of false-negative results associated with testing during early disease and the subsequent possibility of undetectable levels of specific antibodies (window period) [7].

On day 1 (first study visit), eligible patients were recruited to the study, and written informed consent was obtained. An extra blood sample was collected for serological assays (anti-SARS-CoV-2 IgM and IgG) at the same moment of blood collection for routine scheduled tests (no additional venous puncture was needed), and patients were asked to fill in two paper questionnaires (symptoms and epidemiology).

On study days 29-57 (4-8 weeks after the first visit), patients who remained on active treatment with chemotherapy or immunotherapy at our unit had a follow-up anti-SARS-CoV-2 serological test (second study visit). Blood collection was performed at the same moment of blood collection for routine tests, and no additional puncture was needed. Again, patients were asked to fill in two questionnaires (symptoms and epidemiology).

Fourteen days after each study visit, the patients received a follow-up phone call for the assessment of symptoms attributable to COVID-19 and/or diagnosis of COVID-19 in the previous 14 days.

The results of the serological tests were not known at the time of treatment administration. There was no sufficient evidence on interpretation of these results, and therefore, they did not influence patient management.

The study protocol was approved by the Hospital da Luz Lisboa Ethics Committee (reference number: CES/10A/2020/ME).

Study sample

This study was designed to include 100 patients, a convenience sample that reflects the expected accrual of patients in a two- to three-week period, considering the number of patients treated at our Medical Oncology Unit fulfilling the study inclusion criteria. Recruitment started on 6th May 2020 and was planned to continue until patient 100 was included.

The inclusion criteria were as follows: 18 years old or older; ability to understand and sign a written informed consent; diagnosis, documented in the electronic medical record (EMR), of one or more solid or hematologic malignancies, regardless of the clinical or pathological staging; treatment with chemotherapy or immunotherapy (or its combination, with or without other concomitant therapies, including radiation therapy), administered on an outpatient basis, orally or intravenously; and SARS-CoV-2 RNA molecular detection test in a respiratory sample (oropharynx and deep nasopharynx) within a maximum of 30 days prior to study enrolment. Patients were excluded in case of a previous diagnosis of symptomatic COVID-19, presumed or confirmed, if signs or symptoms possibly related to SARS-CoV-2 infection were present in the previous 14 days, or if they were participating in a clinical trial.

Laboratory procedures

IgM and IgG Abs directed against both spike (S) and nucleocapsid (N) SARS-CoV-2 proteins were analyzed using the MAGLUMI 2019-nCoV IgG and IgM (Snibe, Shenzhen, China) chemiluminescence assay (specificity: 97%). In addition, IgG-positive results were subject to confirmation, in the same serum sample, by chemiluminescence assays IgG Abbott Architect SARS-CoV-2 (Abbott Diagnostics, Chicago, USA) (specificity: >99%) and DiaSorin LIAISON SARS-CoV-2 S1/S2 (DiaSorin, Saluggia, Italy) (specificity: 98.5%), detecting antibodies specific for SARS-CoV-2 N and S proteins, respectively.

In chemiluminescence-based assays, the detection of IgG or IgM is based on double-antibody sandwich immunoassay. Recombinant antigens rN and rS were conjugated with fluorescein isothiocyanate (FITC) and immobilized on the anti-FITC antibody-conjugated magnetic particles. Alkaline phosphatase-conjugated human IgG/IgM antibody was used as the detection antibody. An automated magnetic chemiluminescence analyzer was used to read the measured values of chemiluminescence. The results of these tests are presented as arbitrary units [8]. The manufacturer-defined cutoffs for positivity were greater than or equal to 1.00 arbitrary units per mL (AU/mL) for the MAGLUMI 2019-nCoV IgG and IgM, 1.40 signal/cutoff index (index (S/C)) for the IgG Abbott Architect SARS-CoV-2, and greater than or equal to 15.0 AU/mL for the DiaSorin LIAISON SARS-CoV-2 S1/S2.

SARS-CoV-2 RNA molecular detection tests in respiratory samples (henceforth designated by RT-PCR) were routinely performed according to standard clinical practice and not part of study procedures. Therefore, different laboratory methods and timings may have been applied.

Statistical considerations

In this study, the seropositivity of a sample was determined according to the cutoff value defined by the manufacturer of the serological test, with seroprevalence defined as the proportion of seropositive individuals in the sample. Continuous variables are described as mean ± standard deviation or median and interquartile range (IQR), depending on the distribution underlying the data. Normality underlying the data was assessed using the Shapiro-Wilk test. For categorical variables, the results are presented as n (%).

## Results

**Figure 1:**
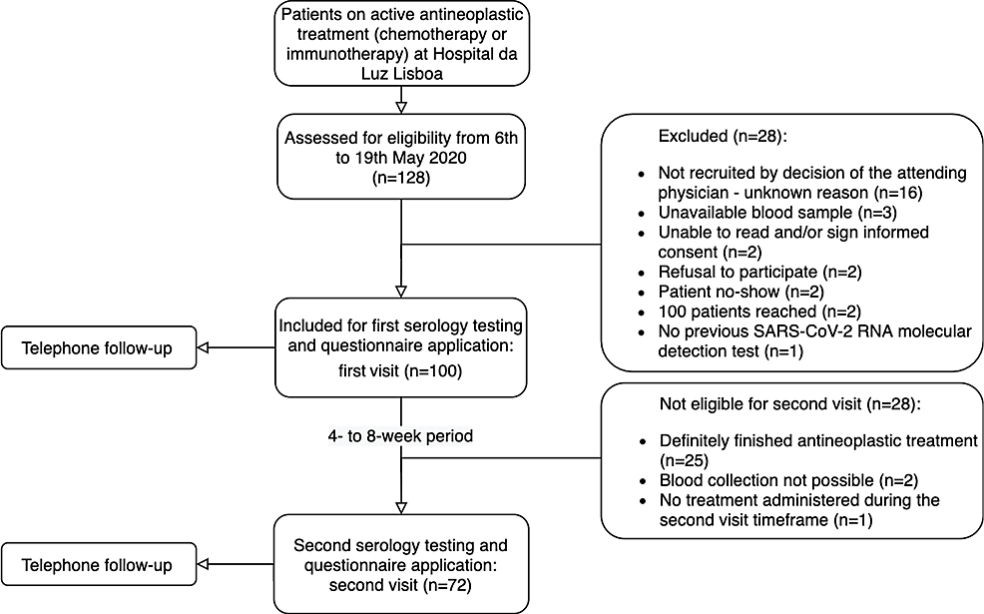
Study design and patient selection flowchart.

One hundred patients were consecutively enrolled during a period of two weeks (6th-20th May 2020) at the Oncology Unit of Hospital da Luz Lisboa (Figure 1).

The median age was 64 (interquartile range (IQR): 16), the majority were female (61%), and 95% had an Eastern Cooperative Oncology Group (ECOG) Performance Status of zero or one. The majority of malignancies were carcinomas (90%), with a vast range of primary locations, including the head and neck, digestive system, lung, breast, genitourinary, and central nervous system and hematological. At the time of the first visit, 56% of the patients were on systemic treatment with curative intent and 44% on palliative treatment (23% first line and 21% second line or subsequent). In addition, 97% were receiving chemotherapy, alone or combined with anti-HER2, anti-VEGF, anti-EGFR, anti-CD20, or proteasome inhibitor, and 3% were being treated with anti-PD-1/PD-L1 (programmed death-1/programmed death ligand-1) alone. Detailed baseline characteristics are presented in the Appendices section.

At the time of the first study visit, no patient had a previously confirmed diagnosis of COVID-19 and only one patient reported previous contact with a COVID-19 patient (61 days before the first study visit). All patients had negative RT-PCR, a median of four days (IQR: 4; minimum: 0; maximum: 29) before the study inclusion. Seventeen patients (17%) were tested more than seven days before the study inclusion because of the following reasons: rescheduling of cancer treatment, without repeating RT-PCR by decision of the attending physician (n=4); urgent change of therapy on the day of inclusion without the possibility of repeating the test (n=1); patient refusal to repeat the test (n=2); patient under single agent anti-PD-1/PD-L1 immunotherapy (n=2); and reason not documented (n=8).

Seventy-two patients attended the second study visit that occurred a median of 42 days (IQR: 7) after the first one. Most patients were on the same antineoplastic treatment regimen as those of the patients in the first visit (n=61, 85%), while the remainder were on a different phase of the same sequential treatment protocol (n=5, 7%) or on a further therapeutic line in the palliative setting (n=6, 8%). None of these patients had a COVID-19 diagnosis within the study timeframe. Between the first and the second study visits, the patients were screened according to standard practice by RT-PCR. The median number of SARS-CoV-2 RNA molecular detection tests performed per patient was two (IQR: 1; minimum: 0; maximum: 5). All tests were negative for the presence of viral RNA. Thirteen patients (18%) were considered non-tested: in three patients (4%), the tests were performed, but not documented on EMR, and 10 patients (14%) were not tested (patient refusal: n=1; patient under isolated anti-PD-1/PD-L1 immunotherapy: n=3; and unknown reason: n=6). For the patient with valid test results, the median time from the last RT-PCR to the second study visit date was two days (IQR: 2.5; minimum: 0; maximum: 56). The reasons for more than seven days between most recent mRNA testing and second visit (n=12) were as follows: delay of treatment, without repeating testing by decision of the attending physician (n=1); patient under single agent anti-PD-1/PD-L1 immunotherapy (n=1); RT-PCR tests performed, but results not documented on EMR (n=4); and unknown reason (n=6).

To the best of our knowledge, none of the remaining 28 patients not eligible for the second study visit had a COVID-19 diagnosis within the study timeframe.

The patients’ self-reported symptoms experienced within the previous 14 days in both study visits are summarized in Table 1. The most frequently reported complaint was tiredness (66% at the first visit and 76% at the second visit), followed by anorexia, myalgia, arthralgia, and dysgeusia. All reported symptoms were considered by the attending physician to be attributable to an alternative diagnosis other than COVID-19.

**Table 1:** Patient self-reported symptoms. *All symptoms were considered by the attending physician to be due to a diagnosis other than COVID-19 infection.

Reported symptoms in previous 14 days (any degree), n (%)*	First study visit valid answers (n=99)	Second study visit valid answers (n=66)
Measured body temperature above 37.5ºC	7 (7)	6 (9)
Cough	14 (14)	10 (15)
Dyspnea (shortness of breath)	5 (5)	4 (6)
Dysgeusia (changes in taste)	28 (28)	31 (47)
Anosmia (loss of smell)	7 (7)	6 (9)
Tiredness	65 (66)	50 (76)
Anorexia (lack of appetite)	22 (22)	22 (33)
Myalgia (muscle pain)	27 (27)	26 (39)
Arthralgias (joint pain)	28 (28)	29 (44)
Odynophagia (sore throat)	7 (7)	9 (14)
Chest pain (chest pain)	8 (8)	6 (9)
Headache	18 (8)	15 (23)
Abdominal pain (bellyache)	16 (16)	14 (21)
Vomiting	12 (12)	13 (20)
Diarrhea	17 (17)	16 (24)

The results of the serological assays performed on both study visits are shown in Table 2.

**Table 2:** Serological assay results (MAGLUMI 2019-nCoV (Snibe)). IgG, immunoglobulin G; IgM, immunoglobulin M

Study visit	Positive, n (%)	Negative, n (%)
First study visit		
IgM	1 (1%)	99 (99%)
IgG	2 (2%)	98 (98%)
Second study visit		
IgM	2 (2.8%)	70 (97.2%)
IgG	0 (0%)	72 (100%)

Two patients had a positive SARS-CoV-2 IgG in the first study visit sample, but this result was not confirmed positive in either of the two IgG confirmatory assays (IgG Abbott Architect SARS-CoV-2 and DiaSorin LIAISON SARS-CoV-2 S1/S2, detecting N and S proteins, respectively) performed in the same serum samples. Both had same-day negative SARS-CoV-2 IgM. One of these patients had a same-day negative SARS-CoV-2 RT-PCR test, and the other had a similar negative test performed four days prior to the serological test. None of them reported previous contact with a known COVID-19 patient.

The patient that had detectable serum IgM had a same-day negative serum IgG and RT-PCR. This patient had no COVID-19 symptoms between the first and the second visits, maintained IgM positivity, had a negative IgG at the second visit (56 days after the first visit), and was tested RT-PCR negative four times within the same timeframe.

A fourth patient tested serum IgM-positive at the second study visit but was IgG-negative and had negative SARS-CoV-2 RT-PCR nasopharyngeal swab on the same day.

No patient had a positive serum IgG at the second visit. One of the patients with detectable IgG at visit one tested negative for both IgM and IgG at study visit two. Unfortunately, there was no blood sample collection on visit two from the other IgG-positive patient at study visit one.

Thus, in this population of 100 patients with cancer undergoing active cancer treatment, we did not document evidence of SARS-CoV-2 infection by either mRNA RT-PCR testing or serological testing.

## Discussion

The first cases of COVID-19 were reported from Wuhan, Hubei, China, in November 2019. On 31st January 2020, Italy reported the first European cases, and the WHO classified SARS-CoV-2 as a pandemic on 11th March 2020 [9]. As of 8th December 2021, more than 266 million cases and 5.27 million deaths have been reported [10]. Thus, in April 2020, when this study was planned, Portugal was on lockdown, and there were significant doubts on the safety of having patients come to the hospital and regarding the safety of administering cancer chemotherapy to patients with cancer with the associated increased risk of infection. As stated before, as of April 2020, the Portuguese Health Authority recommended that patients with cancer be tested by RT-PCR prior to the administration of every cycle of active systemic anticancer treatment, namely, chemotherapy [6]. Thus, it was critical to evaluate the safety of pursuing clinic visits and systemic treatment on patients with cancer. Now, we know that despite the decision to maintain cancer treatment in most patients, COVID-19 resulted in a delay in cancer diagnosis and an increase in non-COVID-19-related deaths [11].

Unequivocal evidence of acute or prior infection by SARS-CoV-2 was not documented in this sample of patients with cancer undergoing antineoplastic treatment. Thus, maintaining active cancer treatment was shown to be the right clinical decision.

Despite two SARS-CoV-2 IgG-positive patients on the first assay, these results were not confirmed on the two confirmatory assays. Since the beginning of the COVID-19 pandemic, multiple serological assays for anti-SARS-CoV-2 antibody detection have been developed and studied. A meta-analysis and systematic review of studies involving antibody tests to detect SARS-CoV-2 infection summarized evidence from 38 studies involving 7,848 individuals and showed very different precision of the available techniques and kits [8]. When our study was designed, the chosen serological assay was the only one available at our hospital laboratory, with manufacturer-claimed clinical sensitivities for IgM and IgG of 78.65% and 91.21%, respectively, and specificities for IgM and IgG of 97.50% and 97.3%, respectively; we found this to be a reliable immunoassay for the measurement of specific IgM and IgG in the sera of COVID-19 patients in a validation study [12].

Given the expected low seroprevalence setting, two different confirmatory assays were used to rule out false-positive IgG results using the same serum sample. The Abbott Architect SARS-CoV-2 IgG reported a specificity of 99.90% and a sensitivity of 100% at day 17 after symptom onset and day 13 after PCR positivity [13]. The DiaSorin LIAISON SARS-CoV-2 S1/S2 IgG test reported a sensitivity of 97.4% 15 days after disease onset and a specificity of 98.5%; however, a study comparing the performance of seven commercially available serological assays reported a sensitivity of 95.5% and a specificity of 90.5% for this assay [14]. Therefore, we believe that in this 100-patient cohort, the two positive IgG results, without a previous known COVID-19 diagnosis or contact with a COVID-19 patient, not confirmed on the confirmatory serological assays, were most likely false-positive results.

As for the two positive IgM results, no evidence of acute or prior COVID-19 infection was found in these patients, and in accordance with the expected assay performance, false positives cannot be excluded. The patient who remained IgM-positive throughout the two study visits, without evidence of IgG seroconversion, suggests interference with the test results.

Other studies conducted during the first wave of the COVID-19 pandemic showed a low seroprevalence in the general population. A study of 17,368 individuals in the city of Wuhan, the epicenter of the COVID-19 pandemic in China, showed that the seropositivity varied between 3.2% and 3.8% and progressively decreased in other cities as the distance to the epicenter increased [15]. In Switzerland, a study conducted in Geneva, a region considered to have a high prevalence of COVID-19 (5,000 reported clinical cases over less than 2.5 months in the population of half a million people) documented a seroprevalence between 4.8% and 10.8% [16]. In Portugal, a general population study of the prevalence of SARS-CoV-2-specific antibodies performed between May and July 2020 recruited 2,301 participants and reported 30 (1.9%) (95% CI: 1.1%-3.1%) IgM-positive and 33 (1.9%) (95% CI: 1.2%-3.1%) IgG-positive individuals [17]. Similar to our study, in a tertiary care cancer center in Austria, 84 patients were tested between 21st March and 4th June 2020 with a reported SARS-CoV-2 IgG seroprevalence of 2.4% (95% CI: 0.3%-8.3%) [18].

Overall, the expected general population seroprevalence was low. In Portugal, at the date of the last study sample collection (14th July 2020), national health authorities had reported 47,051 confirmed COVID-19 cases, approximately 0.5% of the Portuguese population (for a population of ~10.1 million inhabitants). Among patients with cancer, a study reported a significantly lower detection rate of SARS-CoV-2 antibodies 15 days or later after the development of COVID-19 symptoms and RT-PCR-positive testing (versus healthy healthcare workers), more often undetectable in patients who had received cancer treatments in the month before testing [19]. Since patients with cancer are at higher risk of complications from COVID-19 [4,5], it is likely that they have higher individual adherence to isolation and protection measures, leading to less exposure to the virus. Protection measures and isolation of patients with cancer (e.g., at our hospital, the Oncology Unit and Day Hospital were temporarily moved to a separate building to isolate patients with cancer from all other patients) also contributed to the safety of maintaining active cancer treatment.

The limitations of this study include its small sample size and the absent second study visit for 28 of the 100 patients. Since RT-PCR was not part of study procedures, rather performed at the discretion of attending physicians, in accordance with standard clinical practice, these tests were performed at a median of four days (IQR: 2-6) before serological testing; thus, in theory, acute COVID-19 asymptomatic infections could have been missed on the day of blood collection. At that point in time, low antibody concentrations in the early course of the disease could be below the manufacturer’s positive cutoff value of the serological assay [20]. To mitigate this risk, we measured anti-SARS-CoV-2 antibodies at two different time points and did an extensive review of the patients’ EMR and follow-up telephone contacts that documented no cases of COVID-19 infection (symptomatic or asymptomatic) within the study timeframe.

The self-reported symptom questionnaire included symptoms that could be associated not only with COVID-19 diagnosis but also with the oncologic disease or its treatment. A Canadian study polled 4,240 adults about self-reported COVID-19 experience in March 2020 (early in the pandemic) and found that between 5% and 8% of the respondents reported that either themselves or at least one of the household members had possible COVID-19 symptoms [21]. In our study, as expected, patients with cancer reported a higher incidence of symptoms that could be COVID-19 related than the previously mentioned study (general population). However, care must be taken when considering self-reported symptoms, as they are subject to limitations and misclassification.

The presented data is not conclusive regarding the role of anti-SARS-CoV-2 serology in the screening of patients with cancer for COVID-19 active or prior infection in an early phase of the pandemic. However, given the low incidence of COVID-19 in this study, it raises two questions: first, if the changes or delays in the oncologic treatment plan recommended by scientific societies [22], irrespective of local SARS-CoV-2 infection incidence rate, were adequate and what will be the impact on cancer outcomes and, second, if serological testing should be implemented on low incidence settings, given the rate of false positives and the subsequent misinterpretation of the individual immune status.

## Conclusions

COVID-19 was and still is a major public health challenge. However, no additional exposure risk to SARS-CoV-2 was found in this cancer patient cohort compared to results described in general population seroprevalence studies at the time of the first wave of the COVID-19 pandemic. The data reported in this study support the safety of pursuing active cancer treatment despite the restraints of the SARS-CoV-2 pandemic. We suggest not delaying cancer diagnosis and treatment, and with the appropriate constraints, we consider that it is possible to deliver cancer treatment safely.

## Appendices

The detailed baseline characteristics of the patients are presented in Table 3.

**Table 3:** Baseline characteristics. *This patient had a synchronous genitourinary carcinoma on palliative treatment (not chemotherapy or immunotherapy). **Alone or combined with anti-HER2 (n=6), anti-VEGF (n=3), anti-EGFR (n=3), anti-CD20 (n=7), or proteasome inhibitor (n=1). ECOG, Eastern Cooperative Oncology Group; IQR, interquartile range; PD-1/PD-L1, programmed death-1/programmed death ligand-1

Baseline characteristic	Total sample (n=100)
Age, years	
Median	64
IQR	16
Sex, n (%)	
Female	61 (61)
Male	39 (39)
ECOG/Zubrod performance status, n (%)	
0	69 (69)
1	26 (26)
2	5 (5)
Charlson Comorbidity Index (CCI), n (%)	
2–3 points	17 (17)
4–5 points	23 (23)
≥6 points	60 (60)
Smoking status	
Never smokers	44 (44)
Former smokers	39 (39)
Current smokers	6 (6)
Unknown	11 (11)
Malignancy – histology, n (%)	
Carcinoma	90 (90)
Lymphoma	7 (7)
Central nervous system	2 (2)
Multiple myeloma	1 (1) *
Malignancy – primary site, n (%)	
Head and neck	3 (3)
Digestive system	29 (29)
Lung	15 (15)
Breast	35 (35)
Genitourinary	8 (8)
Central nervous system	2 (2)
Hematological	8 (8)
Malignancy – time from diagnosis to the first study visit, months	
Median	5
IQR	15
Systemic treatment intent at the time of the first visit, n (%)	
Curative (neoadjuvant/adjuvant or definitive chemoradiotherapy)	56 (56)
Palliative (first line)	23 (23)
Palliative (second line or subsequent)	21 (21)
Systemic treatment – pharmacological class, n (%)	
Chemotherapy **	97 (97)
Anti-PD-1/PD-L1 alone	3 (3)
Previous surgery (with hospital admission >24 hours), n (%)	
<60 days	9 (9)
60–180 days	25 (25)
None or >180 days	66 (66)
Previous radiotherapy, n (%)	
<60 days	5 (5)
60–180 days	4 (4)
None or >180 days	91 (91)
